# A Vaccine That Co-Targets Tumor Cells and Cancer Associated Fibroblasts Results in Enhanced Antitumor Activity by Inducing Antigen Spreading

**DOI:** 10.1371/journal.pone.0082658

**Published:** 2013-12-12

**Authors:** Stephen Gottschalk, Feng Yu, Minjun Ji, Sunitha Kakarla, Xiao-Tong Song

**Affiliations:** 1 Center for Cell and Gene Therapy, Texas Children's Hospital, Houston Methodist Hospital, Baylor College of Medicine, Houston, Texas, United States of America; 2 Department of Pathology and Immunology, Baylor College of Medicine, Houston, Texas, United States of America; 3 Interdepartmental Program in Translational Biology and Molecular Medicine, Baylor College of Medicine, Houston, Texas, United States of America; 4 Department of Pediatrics, Baylor College of Medicine, Houston, Texas, United States of America; 5 Texas Children's Cancer Center, Texas Children's Hospital, Baylor College of Medicine, Houston, Texas, United States of America; University of Queensland Diamantina Institute, Australia

## Abstract

Dendritic cell (DC) vaccines targeting only cancer cells have produced limited antitumor activity in most clinical studies. Targeting cancer-associated fibroblasts (CAFs) in addition to cancer cells may enhance antitumor effects, since CAFs, the central component of the tumor stroma, directly support tumor growth and contribute to the immunosuppressive tumor microenvironment. To co-target CAFs and tumor cells we developed a new compound DC vaccine that encodes an A20-specific shRNA to enhance DC function, and targets fibroblast activation protein (FAP) expressed in CAFs and the tumor antigen tyrosine-related protein (TRP)2 (DC-shA20-FAP-TRP2). DC-shA20-FAP-TRP2 vaccination induced robust FAP- and TRP2-specific T-cell responses, resulting in greater antitumor activity in the B16 melanoma model in comparison to monovalent vaccines or a vaccine encoding antigens and a control shRNA. DC-shA20-FAP-TRP2 vaccination enhanced tumor infiltration of CD8-positive T cells, and induced antigen-spreading resulting in potent antitumor activity. Thus, co-targeting of tumor cells and CAFs results in the induction of broad-based tumor-specific T-cell responses and has the potential to improve current vaccine approaches for cancer.

## Introduction

Dendritic cell (DC) vaccines have shown limited antitumor activity in the majority of clinical trials [1]. This lack of efficacy is most likely due to the presence of immunosuppressive cells within the tumor as well as the tumor supporting stroma limiting cross presentation and the induction of broad-based tumor antigen-specific T-cell responses [2], [3].

Cancer associated fibroblasts (CAFs) are the key cellular component of the tumor stroma and are present in the majority of common epithelial cancers such as lung, breast, and prostate cancer, as well as sarcoma and melanoma [4]–[7]. CAF mediate resistance to chemo-, radio- and immunotherapy, and CAF tumor content or genes expressed in CAFs correlate with outcome [5], [8]–[10]. CAFs express fibroblasts activating protein (FAP) [11] and the targeted deletion of FAP-positive CAFs in transgenic mouse models has potent antitumor effects highlighting their central role in tumorigenesis [12]. In addition, FAP-targeted vaccines reduced tumor collagen content and modulated the tumor immune microenvironment in preclinical models [13], [14].

We previously have shown that silencing A20, a negative regulator of NF- κb-mediated DC activation, enhances DC function [15]. The intent of this study was now to evaluate a vaccine that silences A20, and co-targets FAP-positive CAFs as well as tumor cells. Our results show that such a compound-vaccine has potent antitumor activity and that co-targeting of CAFs and tumor cells is critical for the induction of cytotoxic T cells specific for tumor antigens not encoded by the vaccine.

## Materials and Methods

### DC immunization and tumor models

This study was approved by the Institutional Animal Care and Use Committees of Baylor College of Medicine (BCM). C57BL/6J were purchased from Jackson Laboratories and maintained in a pathogen-free mouse facility at BCM according to institutional guidelines. DCs were washed in PBS and injected in the rear footpad of naïve C57BL/6 at 1×10^6^ cells/mouse. Mice were sacrificed on indicated days; inguinal lymph nodes and spleens were removed for intracellular staining and Elispot, respectively. For the tumor model, C57BL/6 mice were injected with 5×10^5^ B16, B16-OVA, or EG7-OVA tumor cells subcutaneously. Five days later, mice were randomly divided into groups (n = 5 per groups) and injected with 1×10^6^ lentivirus-transduced LPS-matured DCs. Tumor volumes were measured two or three times per week with a vernier caliper.

### Lentiviral vector construction, production and transduction

The HIV self-inactivating (SIN) vector used in this study was pSIH1-H1-shRNA vector from SBI. Mouse A20 small hairpin interfering RNA sequence was inserted into pSIH1-H1-shRNA to generate pSIH1-H1-A20-shRNA that contains the A20 shRNA (5′- CTACCTGAGTTCCTTCCCCTTCAAGAGAGGGGAAGGAACTCAGGTAGTTTTT-3′). FAP or TRP-2 cDNA were inserted into pSIH1-H1-shRNA under the control of CMV promoter to generate pSIH1-H1-A20-shRNA-CMV-FAP, pSIH1-H1-A20-shRNA-CMV-TRP2, or pSIH1-H1-A20-shRNA-CMV-FAP-TRP2. Recombinant pseudotyped lentiviral vectors were generated as previously described [15] and concentrated by PEG-it™ virus precipitation solution (System Biosciences, Mountain View, CA).

### Dendritic culture

Bone marrow dendritic cells (DCs) were obtained as described [16] with the following modifications. Red blood cells were lysed by incubation at room temperature in Red Blood Cell Lysing Buffer (Sigma-Aldrich, St. Louis, MO) and cells were maintained in HyClone RPMI 1640 supplemented with 10% fetal bovine serum (Summit, Fort Collins, CO), non-essential amino acids, HEPES buffer, glutamax, β-Mercaptoethanol, IL-4 (20 ng/mL), and GM-CSF (20 ng/mL, Peprotech, Rocky Hill, NJ) for 5 days. DCs were then transduced with lentivirus for 8 hours, then cultured for an additional 12–16 hours with LPS.

### Flow cytometry

Flow cytometric analysis of DCs and T cells and were performed as previously described [15], [17]. Inguinal lymph nodes were dissociated and plated in complete RPMI with lentivirus-transduced DCs for an overnight stimulation at 37°C followed by intracellular staining. Stained cells were analyzed on a FACScalibur instrument (Becton Dickinson (BD), Mountain View, CA) using CellQuest software (BD) for all flow-cytometric analyses.

### Enzyme-linked immunosorbent spot (Elispot) assay

Elispot assays of isolated T cells were performed as described previously described [15], [17]. Spleens were dissociated and splenocytes were purified by using MACS CD4 (L3T4) or CD8 (Ly-2) MicroBeads (Miltenyi Biotec, Auburn, CA). Cells were plated in triplicate with OT-II or OT-I peptide (10 ug/mL) or anti-CD3/CD28 for positive control. Plates were incubated overnight, then IFN-γ secretion was assessed (antibodies: MABTECH). The results were evaluated in a blinded fashion by ZellNet Consulting, Inc. (New York, NY) with an automated Elispot reader system, using KS Elispot 4.3 software.

### Cytotoxicity assay

Cytolytic activity of T cells were assessed with a standard chromium release assay, which measures the ability of in vitro–restimulated splenocytes to lyse target cells (20, 50). Splenocytes pooled from immunized mice were restimulated in vitro in RPMI-1640 containing B16-OVA tumor lysate and IL2 for 4–6 days. B16 and B16-OVA cells were labeled with sodium ^51^Cr chromate solution for 60 minutes at 37°C with shaking. Different numbers of effector cells were incubated with a constant number of target cells (5×10^4^/well) in 96-well U-bottomed plates (200 µl/well) for 4 hours at 37°C. The supernatants from triplicate cultures were collected. Percent lysis was calculated as (experimental release – spontaneous release)/(maximum release – spontaneous release)×100.

### Statistical analysis

For statistical analysis, we used Student's t-test with a 95% confidence limit, defined as p<0.05. Results are typically presented as means ± SEM. For animal experiments, 5 mice were planned to detect a large effect size of 2, which provided at least 80% power with 5% type-I error. For the mouse experiments, survival, determined from the time of tumor cell injection, was analyzed by log-rank test.

## Results

### Functional characterization of lentiviral vectors encoding shA20, FAP and TRP2

**Figure 1:**
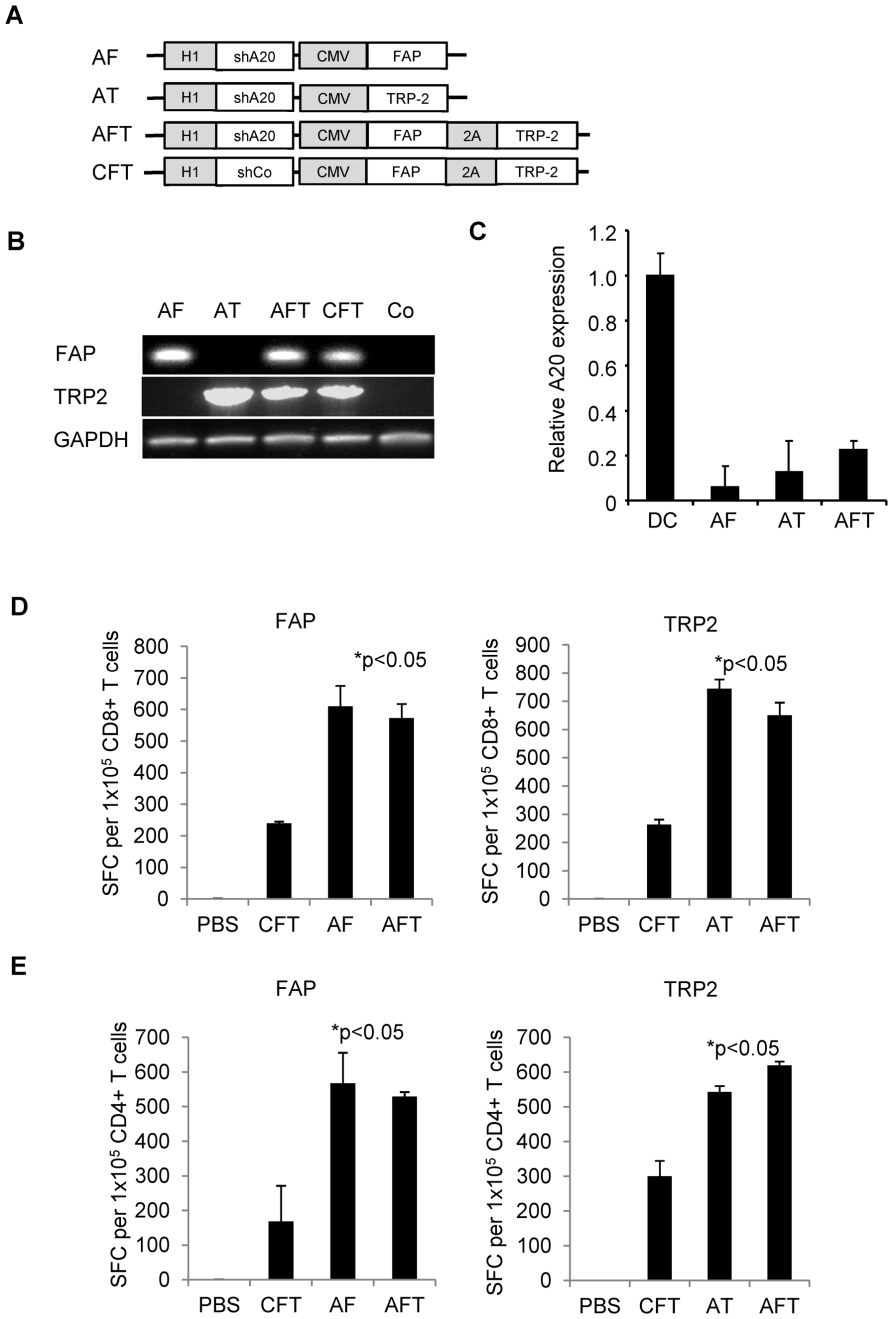
Tumor- and FAP- co-targeted DC induce T-cell activation. (**A**) Scheme of lentiviral constructs. (**B and C**) Mouse BM-DCs were transduced with lentivirus and FAP and TRP2 expression (**B**) and A20 expression (**C**) were detected by RT-PCR or Q-PCR individually. (**D and E**) Mice were immunized with 1×10^6^ lentivirus-transduced BM-DCs in 25 µl sterile PBS or PBS control through footpad 14 days post vaccination splenocytes were prepared and CD8+ (**D**) and CD4+ (**E**) T cells selected. The frequency of FAP- and TRP2-specific T cells was determined using IFN-γ ELISPOT assays (n = 2; assay performed in triplicates). AF, lentiviral vector coexpressing an A20-specific short-hairpin RNA (shRNA) and FAP; AT, lentiviral vector coexpressing A20-shRNA and TRP2; AFT, lentiviral vector coexpressing A20-shRNA, FAP, and TRP2; CFT, lentiviral vector coexpressing GFP-shRNA, FAP, and TRP2; GAPDH, glyceraldehyde 3-phosphate dehydrogenase; PBS, phosphate-buffered saline.

We constructed 4 lentiviral vectors encoding shA20 and FAP (Lv-shA20-FAP), shA20 and TRP2 (Lv-shA20-TRP2), shA20, FAP and TRP2 (Lv-shA20-FAP-TRP2), or a control shRNA, FAP, TRP2 (Lv-shCo-FAP-TRP2) (**Fig. 1A**). Transgene expression, and silencing of A20 was confirmed by RT-PCR in bone-marrow derived DCs transduced with VSV-G pseudotyped lentiviral vectors (**Fig. 1B,C**).

To demonstrate that Lv-transduced DCs induce FAP- and TRP2-specific T-cell responses, mice were vaccinated with 1×10^6^ DC-shA20-FAP, DC-shA20-TRP2, DC-shA20-FAP-TRP2, or DC-shCo-FAP-TRP2. Fourteen days post vaccination CD4- and CD8-positive splenocytes were isolated and the presence of FAP- and TRP2-specific T cells was determined by IFN-γ Elispot assays. Mice vaccinated with A20-silenced DC vaccines induced significantly higher FAP- and TRP2-specific CD4- and CD8-positive T-cell responses (p<0.05) than mice vaccinated with DC-shCo-FAP-TRP2, confirming our previous finding that silencing of A20 in DCs promotes the induction of robust CD4- and CD8-positive antigen-specific T-cell responses *in vivo* (**Fig. 1D,E**) [**15**]. There was no significant difference (p>0.05) in the induction of FAP- or TRP2-specific T cells responses after vaccinating mice with DCs expressing individual (DC-shA20-FAP or DC-shA20-TRP2) or both antigens (DC-shA20-FAP-TRP2), indicating absence of antigenic competition when both antigens are co-expressed.

### DC-shA20-FAP-TRP2 vaccine has potent antitumor activity

**Figure 2:**
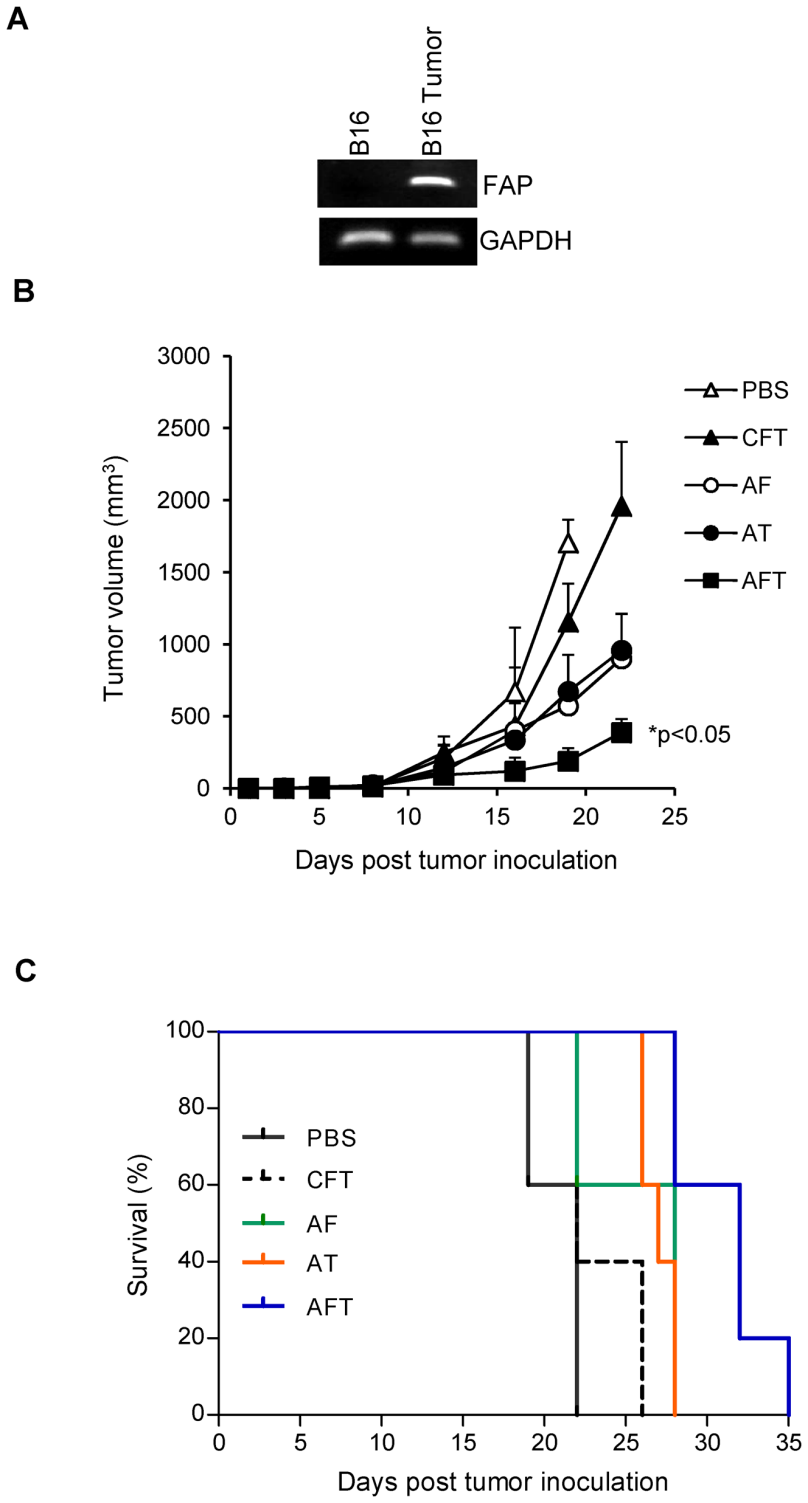
DC-shA20-FAP-TRP2 vaccine has potent antitumor activity. (**A**) RT-PCR for FAP and GAPDH of B16 cell line, and day 5 B16 tumors. (**B,C**) Mice were inoculated with B16 followed by immunization with 1×10^6^ DC-shA20-FAP (AF), DC-shA20-TRP2 (AT), DC-shA20-FAP-TRP2 (AFT), DC-shCo-FAP-TRP2 (ACT) or PBS on day 5 (n =  5 per group). (**B**) Cotargeting FAP and TRP2 with AFT resulted in the greatest antitumor response (AF vs AFT, p<0.05; AT vs AFT, p<0.05). (**C**) Kaplan-Meier survival curve (AF vs AFT, p<0.05; AT vs AFT, p<0.05).

The B16 melanoma model is ideal to evaluate if targeting FAP-positive tumor stroma enhances antitumor effects since B16 cells do not express FAP [18]. We confirmed the induction of FAP expression within 5 days post B16 tumor implantation by RT-PCR (**Fig. 2A**). Mice bearing 5 day-old B16 tumors were vaccinated with a single dose of the 1×10^6^ DC-shA20-FAP, DC-shA20-TRP2, DC-shA20-FAP-TRP2, or DC-shCo-FAP-TRP2 vaccine. All A20-silenced vaccines had antitumor activity, while non-A20 silenced DC vaccine had none (**Fig. 2B**). There was no difference in the antitumor activity of DC-shA20-FAP and DC-shA20-TRP2 vaccines, indicating that T-cell responses against a stroma antigen alone can have significant antitumor effects (**Fig. 2B**). Targeting FAP and TRP2 simultaneously with the DC-shA20-FAP-TRP2 vaccine resulted in the greatest antitumor activity (DC-shA20-TRP2 vs DC-shA20-FAP-TRP2, p<0.05; DC-shA20-FAP vs DC-shA20-FAP-TRP2, p<0.05). This resulted in a significant increase in survival of mice that received DC-shA20-FAP-TRP2 vaccine in comparison to the other vaccine groups (**Fig.2C**). Superior antitumor activity of a tumor- and stroma-targeted vaccine was confirmed using a DC-shA20-FAP-OVA vaccine in B16-OVA and EG7-OVA models (**Fig. S1A,B**).

### DC-shA20-FAP-TRP2 vaccine increases FAP- and TRP2-specific CD8-positive T cells in tumors

**Figure 3:**
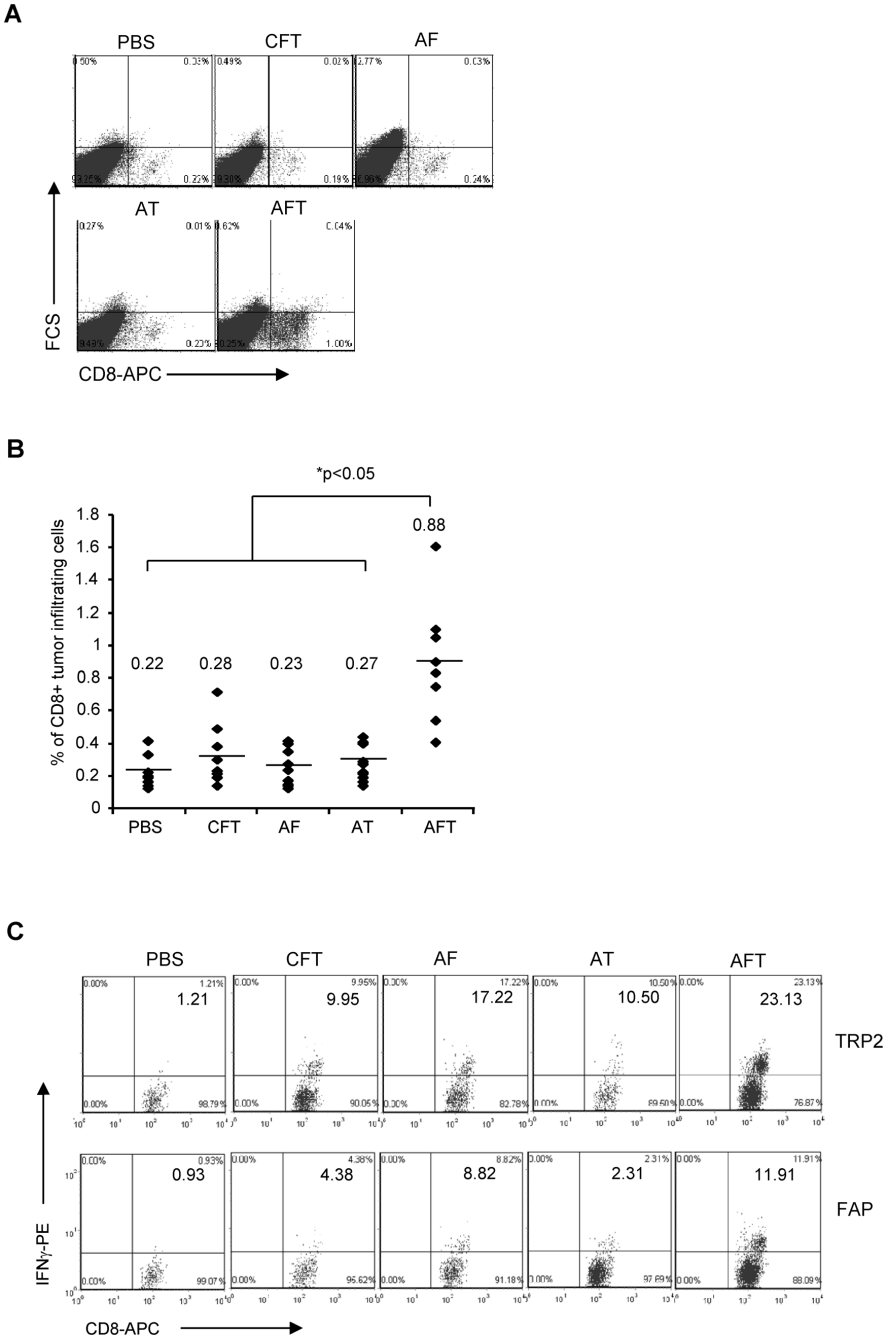
DC-shA20-FAP-TRP2 vaccine increases FAP- and TRP2-specific CD8-positive T cells in tumors. Mice were inoculated with B16 tumors followed by immunization with 1×10^6^ DC-shA20-FAP (AF), DC-shA20-TRP2 (AT), DC-shA20-FAP-TRP2 (AFT) or PBS on day 5. Tumor tissues were dissected 3 weeks post vaccination (n = 5). (**A**) Representative FACS analysis of infiltrating CD8+ T cells. (**B**) Summary data of all mice showing a significant increase (p<0.05) of CD8+ T cells in mice vaccinated with AFT (n = 8 per group). (**C**) TILs were restimulated with DCs transduced with FAP (DC-Lv-FAP) or TRP2 (DC-LV-TRP2) followed by intracellular staining of IFN-γ. One of 2 representative experiments in shown.

To investigate the mechanisms underlying the enhancement of the antitumor effects, we first determined the frequency of CD4- and CD8-positive T cells in B16 tumors 3 weeks post vaccination. While there was no significant difference in the percentage of CD4-positive T cells within tumors between groups of vaccinated mice (data not shown), there was a significant (p<0.05) 4-fold increase in the percentage of CD8-positive T cells after DC-shA20-FAP-TRP2 vaccination in comparison to control mice (**Fig. 3A,B**). In contrast, the monovalent vaccines or the non-silenced FAP/TRP2 DC vaccine did not increase the frequency of intratumoral CD8-positive T cells in comparison to controls. To investigate the specificity of the infiltrating CD8-positive T cells, tumor-infiltrating lymphocytes (TILs) were isolated 3 weeks post vaccination. Intracellular cytokine staining for IFN-γ was performed post stimulation with FAP- or TRP2-expressing DCs. DC-shA20-FAP-TRP2 vaccination induced the highest frequency of FAP- and TRP2-specific T cells in comparison to the other vaccines (**Fig. 3C**), mirroring the previously observed overall increase in the number of CD8-positive T cells within tumors.

### DC-shA20-FAP-TRP2 vaccine induces antigen spreading

**Figure 4:**
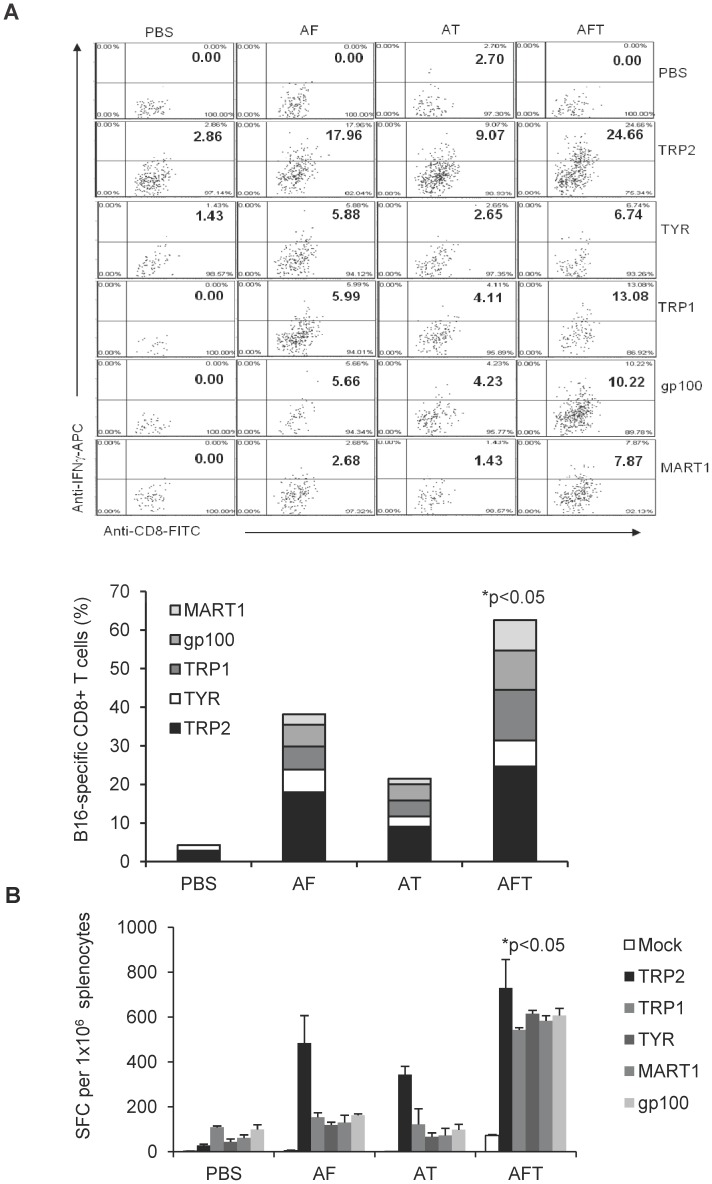
DC-shA20-FAP-TRP2 vaccine induces antigen spreading. Mice were inoculated with B16 tumors followed by immunization with 1×10^6^ DC-shA20-FAP (AF), DC-shA20-TRP2 (AT), DC-shA20-FAP-TRP2 (AFT) or PBS on day 5. TILs and Splenocytes were prepared 3 weeks post vaccination (n = 5). (**A**) TILs were restimulated with DC-Lv-TRP2, DC-Lv-TRP1, DC-Lv-TYR, DC-Lv-gp100, DC-Lv-MART1 or mock transduced DCs (PBS) followed by intracellular staining of IFN-γ. FACS analysis and a cumulative bar graph are shown. TILs isolated from AFT vaccinated mice had the highest frequency of B16-speciifc T cells (p<0.05). (**B**) Splenocytes were subjected to IFN-γ Elispot assays. DC-Lv-TRP2, DC-Lv-TRP1, DC-Lv-TYR, DC-Lv-gp100, DC-Lv-MART1 or mock transduced DCs (PBS) were used as APCs. There was a significant increase (p<0.05) of T cells specific for TRP1, TYR, gp100, and MART1 only in AFT vaccinated mice.

In the previous experiment we noticed that the DC-shA20-FAP vaccine induced TRP2-specific T-cell responses (**Fig. 3C**), suggestive of epitope spreading and the induction of antigen spreading. To further investigate this finding, B16 tumor-bearing mice were vaccinated on day 5 with DC-shA20-FAP, DC-shA20-TRP2, DC-shA20-FAP-TRP2, or PBS. After 3 weeks the frequency of T cells specific for the B16-associated tumor antigens tyrosinase-related protein 1 (TRP1), tyrosinase (Tyr), gp100, and melanoma-associated antigen recognized by T cells (MART1) among splenocytes and tumor infiltrating lymphocytes was determined. While all vaccines induced a significant increase of TRP1-, Tyr-, gp100-, and MART1-specific T cells in TILs in comparison to PBS-injected mice (**Fig. 4A**), the frequency of melanoma-specific T cells was highest in DC-shA20-FAP-TRP2 vaccinated mice (p<0.05). Only the DC-shA20-FAP-TRP2 vaccine induced systemic T-cell responses against TRP1, Tyr, gp100, and MART1 (**Fig.4B**). These results indicate that targeting of the tumor stroma as well as the tumor enables the induction of antigen spreading.

### Antigen spreading results in enhanced antitumor activity

**Figure 5:**
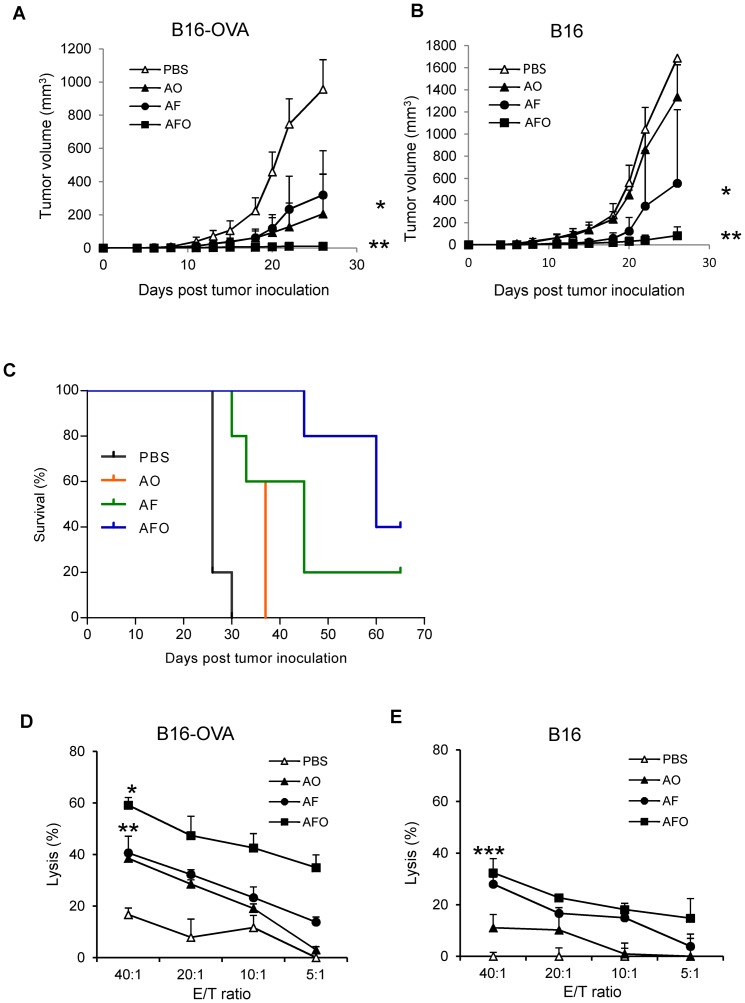
Induced T cells specific for melanoma antigens not present in the vaccine have antitumor activity. (**A-C**) Mice were inoculated with B16-OVA or B16 tumor on the right or left flank separately followed by immunization with 1×10^6^ DC-shA20-FAP (AF), DC-shA20-OVA (AO), DC-shA20-FAP-OVA (AFO) or PBS 5 days later (n = 5 per group). B16-OVA (**A**) and B16 (**B**) tumor growth was measured. The AFO and the AF vaccine had anti-B16 activity with AFO being superior (p<0.05). (**C**) Kaplan-Meier survival curve (AO vs AF, p<0.05; AO vs AFO, p<0.05). (**D,E**) Mice were inoculated with B16-OVA followed by immunization with 1×10^6^ AF, AO, AFO, and PBS 5 days later. 3 weeks later, splenocytes were prepared and cultured in vitro in the presence of B16-OVA tumor lysate and IL2 for 5 days and their cytolytic activity was evaluated in standard subjected ^51^Cr release assay against B16-OVA (**D**) or B16 (**E**). Splenocytes isolated from AF and AFO vaccinated mice had significant cytolytic activity B16. *AFO vs AF and AO, p<0.05; **AF and AO vs PBS, p<0.05;***AFO and AF vs AO, p<0.05).

Having shown that the developed compound vaccines induced B16 antigen spreading we wanted to determine if these antigen-specific T cells have anti-tumor activity. Mice were inoculated with B16-OVA and B16 on their right or left flanks, and after 5 days vaccinated with DC-shA20-FAP, DC-shA20-OVA, DC-shA20-FAP-OVA, or PBS. DC-shA20-OVA vaccination only inhibited the growth of B16-OVA, while the DC-shA20-FAP vaccine inhibited B16 as well as B16-OVA tumor growth (**Fig. 5A,B**). The DC-shA20-FAP-OVA vaccine had the greatest antitumor activity against both tumor cell lines (p<0.05; **Fig. 5A,B**). This is consistent with our observation that co-targeting tumor antigen and FAP induces systemic T cells responses against tumor antigen not present in the vaccine (**Fig.4B**). While DC-shA20-OVA vaccination did not result in an increase in overall survival, due to the antigen negative B16 tumors, DC-shA20-FAP or DC-shA20-FAP-OVA vaccination resulted in a significant increase in overall survival in comparison to DC-shA20-OVA (p<0.05; **Fig 5.C**).

To further confirm the systemic induction of T cells specific for antigens not present in the vaccine, mice were injected with B16-OVA and vaccinated after 5 days with DC-shA20-FAP, DC-shA20-OVA, DC-shA20-FAP-OVA, or PBS. After 3 weeks splenocytes of vaccinated mice were isolated and restimulated for 5 days *ex vivo* before performing a cytotoxicity assay with B16-OVA and B16 cells as targets. Splenocytes harvested from DC-shA20-FAP or DC-shA20-FAP-OVA vaccinated mice showed significant killing of B16-OVA and B16 cells (**Fig. 5D,E**), where as the DC-shA20-OVA vaccine primarily induced OVA-specific T-cell responses.

## Discussion

Our results demonstrate that a DC vaccine in which the antigen presenting attenuator A20 is inhibited, and that targets FAP-positive CAFs and the tumor antigen TRP2 has potent antitumor effects, enables cross-presentation of tumor antigens by intratumoral APCs resulting in the broad-based induction of T cells specific for tumor antigens not included in the vaccine.

The tumor microenvironment is a complex milieu and plays an essential role in tumor initiation, progression and mediating therapeutic resistance [9], [10]. Targeting non-malignant cells present within tumors in addition to cancer cells may therefore increase the effectiveness of cancer-targeted therapies [7], [19], [20]. FAP-positive CAFs, the central cellular component of the tumor stroma, have emerged as key players in promoting extracellular matrix (ECM) remodeling, vascularization and immunosupression [21]. For example, in transgenic mice, in which the diphtheria toxin receptor is expressed under the control of the FAP promoter, administration of diphtheria toxin, which only kills tumor-associated FAP-positive stromal cells, resulted in complete ablation of solid tumors [12].

FAP is a membrane bound aminopeptidase, which is involved in ECM remodeling [22]. Inhibition of the aminopeptidase activity of FAP resulted in decreased tumor growth and was associated with an accumulation of collagen, decreased numbers of myofibroblasts, and decreased tumor vascularization in preclinical models [21], however no objective clinical responses were observed in clinical trials [23]. Targeting FAP with a humanized monoclonal antibody, sibrotuzumab, also showed no objective clinical responses; however, imaging studies revealed that the antibody preferentially localized to metastatic tumor sites after administration [24].

Several groups have conducted vaccine studies solely targeting FAP, demonstrating that S. typhimuirum-, plasmid-, or DC-based FAP vaccines have antitumor activity in preventive or early therapeutic settings, modulate the tumor microenvironment by promoting Th1 polarization and enhancing the infiltration of CD8-positive T cells [13], [14], [18], [25]. While cotargeting of FAP-positive CAFs and tumor cells with a DC vaccine showed a significant delay in tumor growth in one preclinical study, no mechanistic studies were performed [18].

Our studies now extend these findings and demonstrate that silencing the ubiquitin ligase A20, an antigen presenting attenuator in DCs, results in a significant increase in the frequency of FAP-specific T cells in comparison to a DC vaccine with a control shRNA. This highlights the important role of silencing negative regulators such as A20 in APCs to induce T-cell responses against self-antigens such as FAP [15]. We observed no difference in the antitumor activity of DC-shA20-FAP and DC-shA20-TRP2, indicating that targeting CAFs by FAP vaccination can induce antitumor effects similar to vaccines that target malignant cells. However, vaccination with DC-shA20-FAP-TRP2 resulted in the greatest antitumor activity. Mechanistic studied revealed that cotargeting of CAFs and tumor cells resulted in an increased percentage of CD8-positive T cells within tumors and the induction of T cells specific for antigens not present in the vaccine with potent antitumor activity. DC-shA20-FAP, DC-shA20-TRP2, and DC-shCo-FAP-TRP2 vaccine induced comparable levels of CD8-positice TILs (**Fig. 3B**), however only DC-shA20-FAP and DC-shA20-TRP2 vaccines had antitumor activity (**Fig. 2B**). We had previously shown that A20-siRNA-adjuvanted DC vaccines induce potent tumor antigen-specific cytotoxic T cells and T helper cells that are refractory to Treg inhibition [15]. Thus, while all three vaccines induced comparable TIL responses, DC-shA20-FAP and DC-shA20-TRP2-induced TILs are most likely more effective in inhibiting tumor growth.

Vaccine studies in breast cancer, renal carcinoma, and melanoma patients suggest that cross-presentation and epitope spreading correlates with improved overall survival [26]–[28]. In addition, the induction of broad-based tumor-specific T-cell responses after adoptive T-cell transfer in one melanoma patient resulted in a complete response [29]. While these studies highlight the importance of epitope spreading, the determinants of effectively inducing cross-presentation and epitope spreading are currently ill defined. While all vaccines induced low levels of TILs that were specific for antigens not present in the vaccine, only the tumor- and stroma-targeted vaccine induced systemic T-cell responses to all tested non-vaccine antigens. Targeting only the tumor stroma with DC-shA20-FAP resulted in the induction of TRP2-specific TILs and systemic TRP2-specific T-cell responses that were higher (although not significant) in comparison to the DC-sh-TRP2 vaccine. These results suggest that targeting the FAP-positive stroma results in the destruction of B16 cells and reversal of the immunosuppressive tumor microenvironment.

Importantly, the induced tumor-specific T cells were cytotoxic and had antitumor activity against antigen-loss variants (ALVs), which already have been observed in patients enrolled on vaccine or T-cell immunotherapy studies that only target tumor antigens [30], [31]. While other groups have shown in preclinical models that cross presentation of antigens on stromal cells is critical for the elimination of ALVs using a highly expressed model antigen [32], our results indicate that targeting stromal cells is necessary to induce broad-based anti-tumor responses, which should minimize the risk of ALVs.

In summary, the compound vaccine we have developed induces CAF- and tumor-specific immune responses and elicits broad-based T-cell responses against tumor antigens. Thus, targeting CAFs in addition to malignant cancer cells has the potential to improve current vaccine approaches for cancer.

## Supporting Information

Figure S1 DC-shA20-FAP-OVA vaccine has potent antitumor activity.
(PDF)
