# Supplementary material for: A Vaccine That Co-Targets Tumor Cells and Cancer Associated Fibroblasts Results in Enhanced Antitumor Activity by Inducing Antigen Spreading
Source: PLoS One. 2013 Dec 12;8(12):e82658. doi: 10.1371/journal.pone.0082658 (PMC3861387; doi:10.1371/journal.pone.0082658)
Supplement: Figure S1 — DC-shA20-FAP-OVA vaccine has potent antitumor activity. (PDF) [file pone.0082658.s001.pdf]

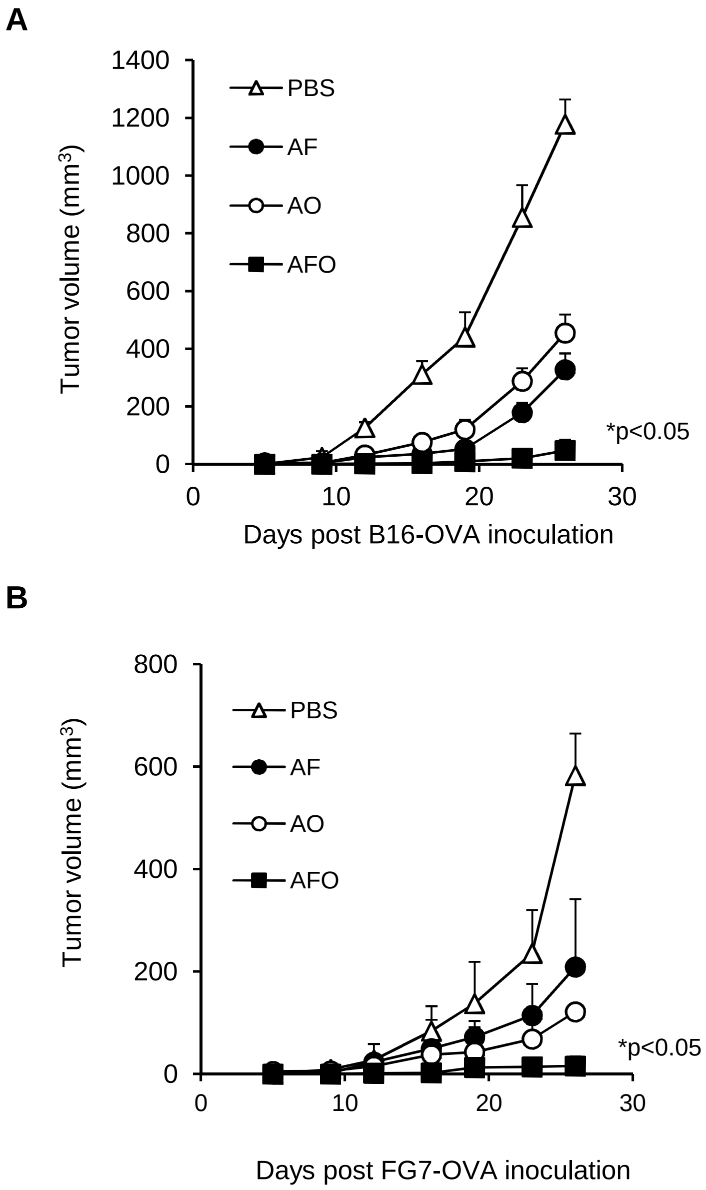

Figure S1: DC-shA20-FAP-OVA vaccine has potent antitumor activity. Mice were inoculated with B16-OVA **(A)** or EG7-OVA **(B)** followed by immunization with $1x10^6$ DC-shA20-FAP (AF), DC-shA20-OVA (AO), DC-shA20-FAP-OVA (AF) or PBS on day 5 (n= 5 per group). Cotargeting FAP and OVA with AFO resulted in the greatest antitumor activity in both models (B16-OVA: AF vs AFO, $p<0.05$; AO vs AFO, $p<0.05$; EG7-OVA: AO vs AFO, $p<0.05$; AF vs AFO, $p<0.05$).
